# Maternal Glucocorticoid Elevation and Associated Fetal Thymocyte Apoptosis are Involved in Immune Disorders of Prenatal Caffeine Exposed Offspring Mice

**DOI:** 10.1038/s41598-017-14103-7

**Published:** 2017-10-23

**Authors:** Han-xiao Liu, Ting Chen, Xiao Wen, Wen Qu, Sha Liu, Hui-yi Yan, Li-fang Hou, Jie Ping

**Affiliations:** Department of Pharmacology, Wuhan University School of Basic Medical Sciences, Wuhan, 430071 China

## Abstract

Our previous study showed that prenatal caffeine exposure (PCE) could induce intrauterine growth retardation (IUGR) and glucocorticoid elevation in the fetus. Researchers suggested that IUGR is a risk factor for T helper cell (Th)1/Th2 deviation. However, whether PCE can induce these immune disorders and the underlying mechanisms of that induction remain unknown. This study aimed to observe the effects of PCE on the Th1/Th2 balance in offspring and further explore the developmental origin mechanisms from the perspective of glucocorticoid overexposure-induced thymocyte apoptosis. An IUGR model was established by caffeine administration from gestational day (GD) 9 to GD 18, and the offspring were immunized on postnatal day (PND) 42. The results show that maternal glucocorticoid overexposure increased fetal thymocyte apoptosis by activating both the Fas-mediated and the Bim-regulated apoptotic pathways. After birth, accelerated thymocyte apoptosis and Th1 suppression were also found in the PCE offspring at PND 14 and PND 49. Moreover, the PCE offspring showed immune disorders after immunization, manifesting as increased IgG1/IgG2a ratio and IL-4 production in the serum. In conclusion, PCE could induce fetal overexposure to maternal glucocorticoids and increase thymocyte apoptosis, which could persist into postnatal life and be implicated in Th1 inhibition and further immune disorders.

## Introduction

Caffeine is a xanthine alkaloid widely found in coffee, tea, soft beverages, food and some analgesic drugs. Because of its effects on stimulating the central nervous system and relieving fatigue, caffeine-containing food and beverages are favored by a large number of people, including pregnant women^1,2^. Epidemiological studies have revealed that caffeine is consumed daily by many pregnant women in developed countries and that caffeine ingestion during pregnancy can cause developmental toxicities, including intrauterine growth retardation (IUGR)^3,4^. Prenatal caffeine exposure (PCE)-induced IUGR models have been successfully established in our previous studies. In addition, programmed alterations in multi-organ development as well as increased susceptibility to adult diseases in offspring have been observed in these PCE models^5,6^. Additionally, rising epidemiological evidence also supports that IUGR is related to a greater susceptibility to some adult immune and inflammatory disorders, especially allergic diseases^7,8^. These studies indicated that PCE is a risk factor for postnatal immune diseases.

Inflammatory and immune disorders, especially allergic diseases, have become a growing health problem worldwide^9–11^. The balance between the capacity of the interferon-gamma (IFN-γ)-mediated T helper cell (Th)1 response and the interleukin (IL)-4-mediated Th2 response is necessary to maintain immune homeostasis following antigen challenge, and the Th1/Th2 deviation appears to be a potential mechanism of allergic diseases^12^. In the fetus, there is a developmentally programmed skewing to the Th2-biased immune phenotype, which is critical for maintaining immunological self-tolerance and protecting the developing embryo from maternal immune responses^13^. Thus, increases in Th1 development and Th1 response capacity after birth are essential for Th1/Th2 balance. Recent research has revealed that prenatal exposure to adverse factors might interrupt the Th1 increase and result in a long-lasting Th2-biased immune phenotype after birth. In addition, this phenotype appears to contribute to an increased risk of immune disorders^13,14^. Therefore, the failure of offspring to increase Th1 development could be closely related to immune disease susceptibility. However, whether PCE can inhibit Th1 development and the underlying mechanisms remains unknown.

Th1 is differentiated from the CD4^+^ single positive (SP) thymocytes. The well-developed and tightly controlled fetal thymopoiesis in utero is critical for the production of CD4^+^ SP thymocytes and the increase in Th1 cells after birth^15–17^. Thymopoiesis is one of the earliest immune processes in the fetus^18^. Based on the expression of CD4 and CD8, the development progress of thymocytes during thymopoiesis can be divided into several subpopulations: CD4^−^ CD8^−^ double negative thymocytes (DN), CD4^+^ CD8^+^ double positive thymocytes (DP) and single positive thymocytes (CD4^+^ SP or CD8^+^ SP). All of these subpopulations express the glucocorticoid receptor (GR)^19,20^. It is known that endogenous glucocorticoids (GCs) can regulate thymocyte development and apoptosis *via* the GR during beta-selection, positive-selection, and negative-selection^19,21^. GCs can induce T lymphocyte apoptosis by two distinct apoptotic pathways. One, also known as the ‘intrinsic’ apoptotic pathway, is activated by the release of apoptotic mediators from the mitochondria following disruption of its outer membrane. The other pathway, also known as the ‘extrinsic’ apoptotic pathway, is initiated by the ligation of death receptors as typified by Fas. These two pathways share common components: a series of cysteine-dependent aspartate-directed proteases (caspases)^22,23^. It has been reported that prenatal exposure to dexamethasone, a synthetic GC, can increase thymocyte apoptosis and attenuate thymopoiesis^24,25^. Other evidence also links maternal GC elevation with abnormal fetal thymocyte development^21^. The results from our previous studies and from other laboratories reported that PCE can elevate GC levels in the fetus^26–28^. Therefore, we speculated that PCE can induce thymocyte apoptosis and attenuate thymopoiesis *via* GCs.

The aim of this study was to investigate the effects of PCE on the Th1/Th2 balance and the immune responses in offspring from the perspective of restricted fetal thymopoiesis, and further, to explore the underlying mechanisms by determining the gene expression of GC-mediated apoptosis pathways in the fetal thymus. This study provides evidence for elucidating the toxicity of PCE on fetal thymocyte development and for exploring the developmental origin of immune disorder susceptibility of IUGR offspring caused by PCE.

## Results

### PCE inhibited offspring development

**Figure 1:**
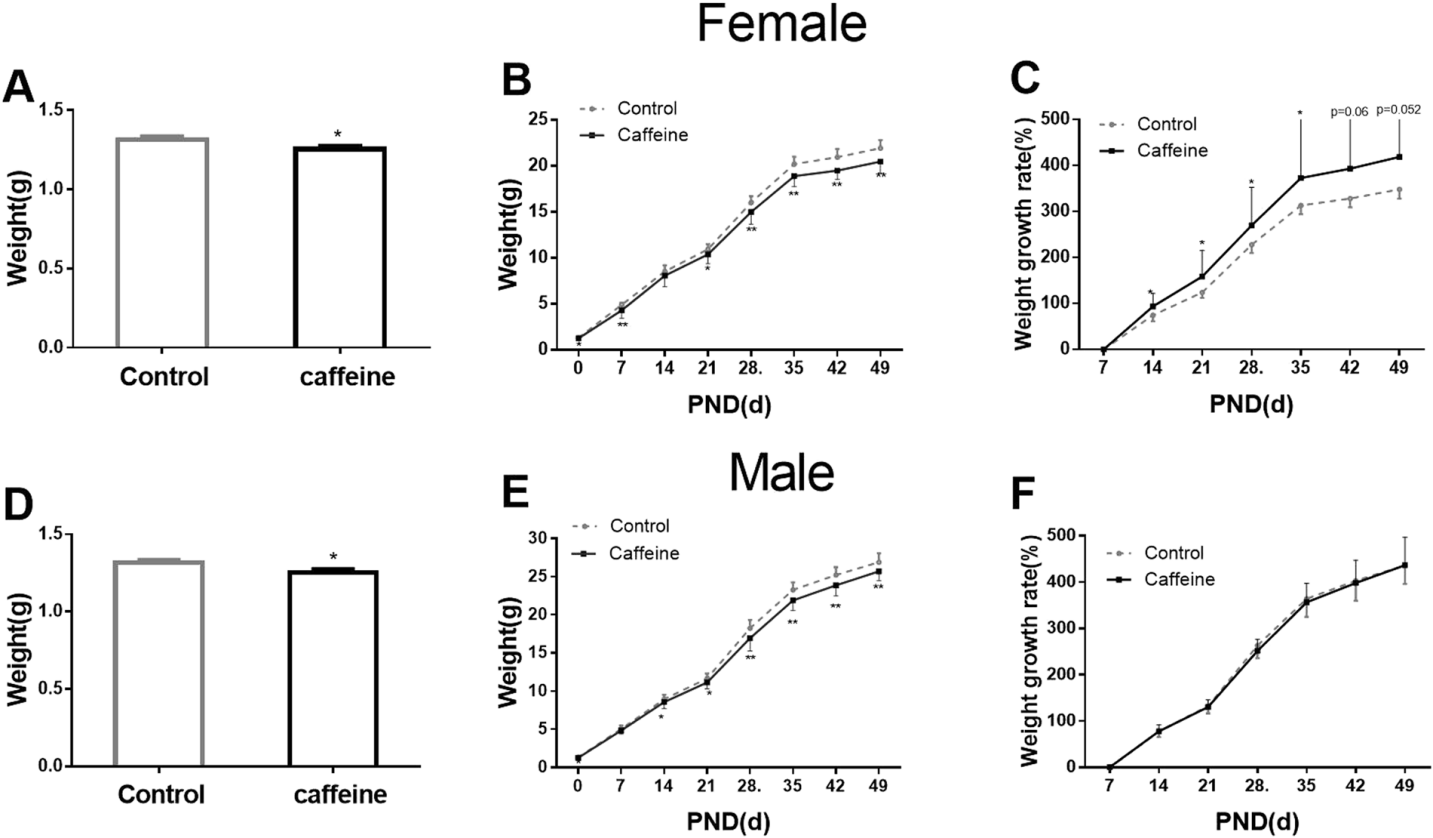
Effects of prenatal caffeine exposure (PCE) on body weight and growth rates of offspring. (**A**,**D**) Offspring body weights on postnatal day (PND) 0; (**B**,**E**) Changes in offspring body weights; (**C**,**F**) Offspring body weight growth rates. Mean ± SD, n = 20 per group per gender. *P < 0.05, **P < 0.01 *vs* control.

Body weight is an important index to reflect offspring development. As shown in Fig. 1A and D, PCE significantly decreased the body weight of both female and male offspring on postnatal day (PND) 0 (*P* < 0.05), and this impact persisted into PND 49 (*P* < 0.05, *P* < 0.01, Fig. 1B and E). Additionally, PCE increased the body weight growth rate in female offspring (*P = *0.06, *P = *0.052, *P* < 0.05, Fig. 1C).

### PCE changed the cytokine expression and production in the offspring on PND 49

**Figure 2:**
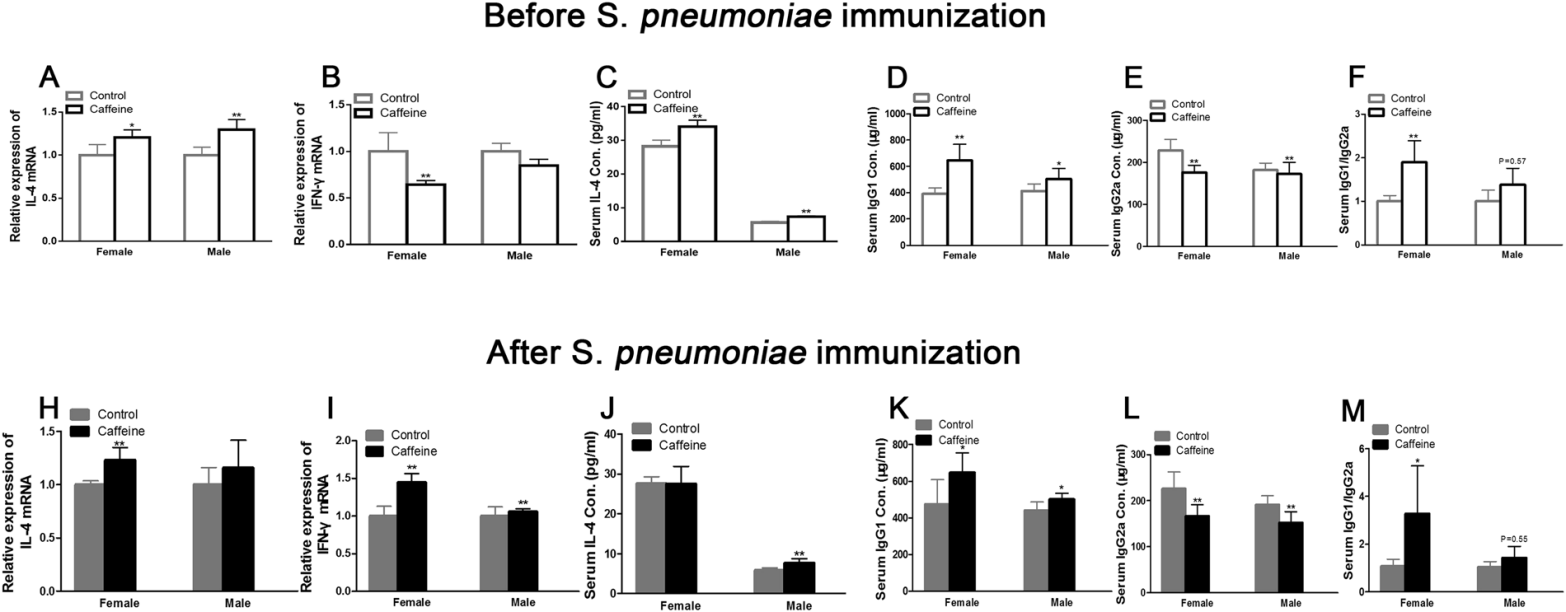
Effects of prenatal caffeine exposure (PCE) on splenic IL-4 and IFN-γ mRNA expression levels, on serum IL-4, IgG1 and IgG2a levels, and on IgG1/IgG2a ratios in the offspring on postnatal day (PND) 49 before and after *S. pneumoniae* immunization. (**A**,**H**) Relative IL-4 mRNA expression levels in the offspring; (**B**,**I**): Relative IFN-γ mRNA expression levels in the offspring; (**C,J)** Serum IL-4 contents in the offspring; (**D**,**K**) Serum IgG1 concentrations in the offspring; (**E**,**L**) Serum IgG2a concentrations in the offspring; (**F**,**M**) IgG1/IgG2a ratios in the offspring. Mean ± SD, n = 8–9 per group per gender. *P < 0.05, **P < 0.01 *vs* control.

IFN-γ and IL-4 are the main representative effector cytokines of Th1 and Th2 cells, respectively. Therefore, the expression of IFN-γ and IL-4 were used in the Th1/Th2 paradigm to reflect immune status. Thus, we detected the IL-4 content in the serum and IFN-γ and IL-4 mRNA expression in the spleen on PND 49. As shown in Fig. 2A and C, significant increases in the serum IL-4 concentration and splenic IL-4 expression were observed in both genders in the PCE group (*P* < 0.05*, P* < 0.01). The IFN-γ mRNA expression levels in the female PCE offspring before immunization were significantly decreased (*P* < 0.01, Fig. 2B) compared with the controls. Additionally, the IFN-γ expression levels in the male PCE offspring were also lower than the controls (Fig. 2B).

To examine the effects of PCE on inflammation development of offspring, offspring were immunized with *Streptococcus pneumoniae (*S*. pneumoniae)*. As shown in Fig. 2J, after immunization, there was no significant difference in the serum IL-4 content between the female PCE offspring and the controls; however, there was a remarkable increase in the serum IL-4 level in the PCE male offspring (*P* < 0.01) compared to controls. Likewise, the splenic IL-4 mRNA expression in the female PCE offspring was notably increased (*P* < 0.01), and an increased tendency was also observed in the PCE male offspring (Fig. 2H). PCE also increased the IFN-γ expression in both the female and male offspring (*P* < 0.01, Fig. 2I).

### PCE attenuated the Th1 response in the offspring following antigen challenge

In mice, Th1 immune responses produce immunoglobulin G (IgG) 2a, and Th2 immune responses promote IgG1 production. An increased IgG1/IgG2a ratio usually reflects a Th1-inhibition phenotype. As shown in Fig. 2D and K, before and after immunization, the serum IgG1 levels in both the male and female PCE offspring were increased (*P* < 0.05) compared with the controls. Additionally, a significant decrease in the serum IgG2a content was observed in the PCE offspring (*P* < 0.01, Fig. 2E and L). As a result, all PCE offspring (before and after immunization) exhibited higher ratios of IgG1/IgG2a compared with the controls (Fig. 2F and M).

### PCE altered the thymocyte phenotypes in postnatal life

**Figure 3:**
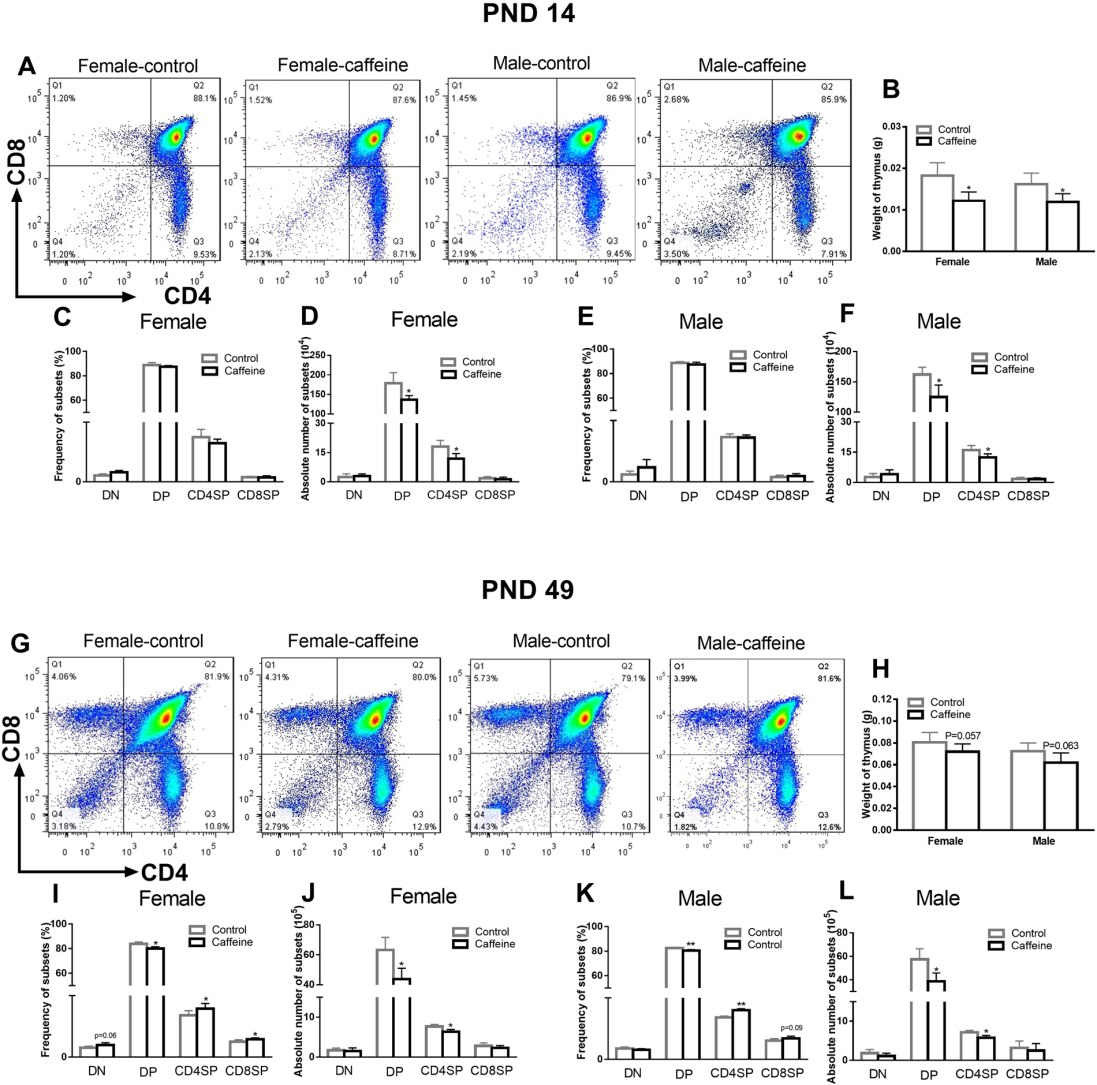
Effects of prenatal caffeine exposure (PCE) on thymocyte phenotypes in the offspring mice on postnatal days (PND) 14 and 49. (**A**,**G**) Typical flow diagrams of male and female offspring; (**B,H**) Thymus weights of the offspring; (**C,I**) Thymocyte subpopulation percentages in the female offspring; (**D,J**) Absolute thymocyte subpopulation numbers in the female offspring; (**E,K**) Thymocyte subpopulation percentages in the male offspring. (**F,L**) Absolute thymocyte subpopulation numbers in the male offspring. Mean ± SD, n = 3 per group per gender. *P < 0.05, **P < 0.01 *vs* control.

As PCE altered the T cell immunity before S. *pneumoniae* challenge, we examined the effects of PCE on thymopoiesis in the offspring during postnatal life. Thymuses were taken and weighted on PND 14 and 49, and then the thymocyte phenotypes were analyzed by flow cytometry. The results showed that PCE significantly decreased the thymus weights on PND 14 (*P* < 0.05, Fig. 3B) compared with the controls. Moreover, the thymus weights of the PCE offspring on PND 49 also showed a decreasing trend (*P* = 0.063, *P* = 0.057, Fig. 3H). As shown in Fig. 3C and E, in both genders, the thymocyte subpopulation percentages were not significantly different between the PCE offspring and the controls on PND 14. On PND 49, the female PCE offspring exhibited higher percentages of CD4SP and CD8SP cells and lower DP cell percentages than the controls (*P* < 0.05, Fig. 3I). Similar results were observed in the male offspring (*P* = 0.09, *P* < 0.01, Fig. 3K). The results also showed that the absolute number of DP and CD4SP thymocytes were significantly decreased in the PCE offspring on PND 14 and on PND 49 (*P* < 0.05, Fig. 3D,F,J, and L). The reason for the discrepancy between the percentages and the absolute numbers of the thymocyte subpopulations may be attributed to thymic weight loss.

### PCE increased thymocyte apoptosis during postnatal life

**Figure 4:**
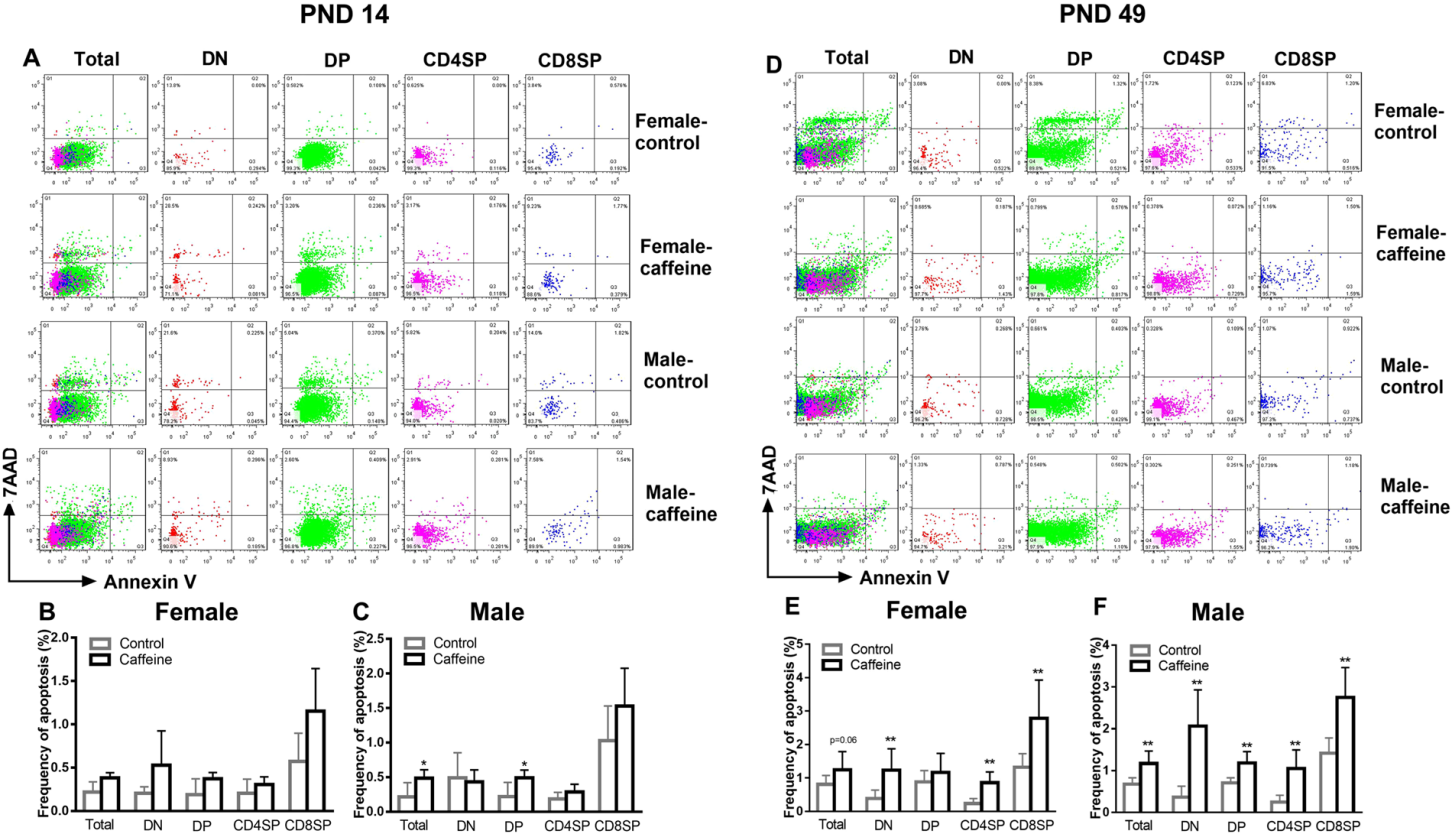
Effects of prenatal caffeine exposure (PCE) on thymocyte apoptosis in offspring mice on postnatal days (PND) 14 and 49. (**A,D**) Typical flow diagrams of thymocyte apoptosis in the female and male offspring (**B,E**) Thymocyte apoptosis percentages in the female offspring; (**C**,**F**) Thymocyte apoptosis percentages in the male offspring. Mean ± SD, n = 3 per group per gender. *P < 0.05, **P < 0.01 *vs* control.

Due to the crucial role of apoptosis in every developmental stage of thymocytes, we further examined thymocyte apoptosis during postnatal life. On PND 14, in both genders, the apoptosis percentages of the total, DP, CD4SP, and CD8SP thymocytes of the PCE offspring were higher than that of the controls (Fig. 4A–C). As the offspring matured, the proportion of thymocyte apoptosis was enhanced in the PCE group. There were significantly increased apoptosis percentages in the total thymocytes and in each subpopulation in the male PCE offspring compared with the controls on PND 49 (*P* < 0.01, Fig. 4F). Similar results were found in the females (*P* < 0.01, Fig. 4E).

### PCE induced IUGR and elevated GCs in the fetal serum

**Figure 5:**
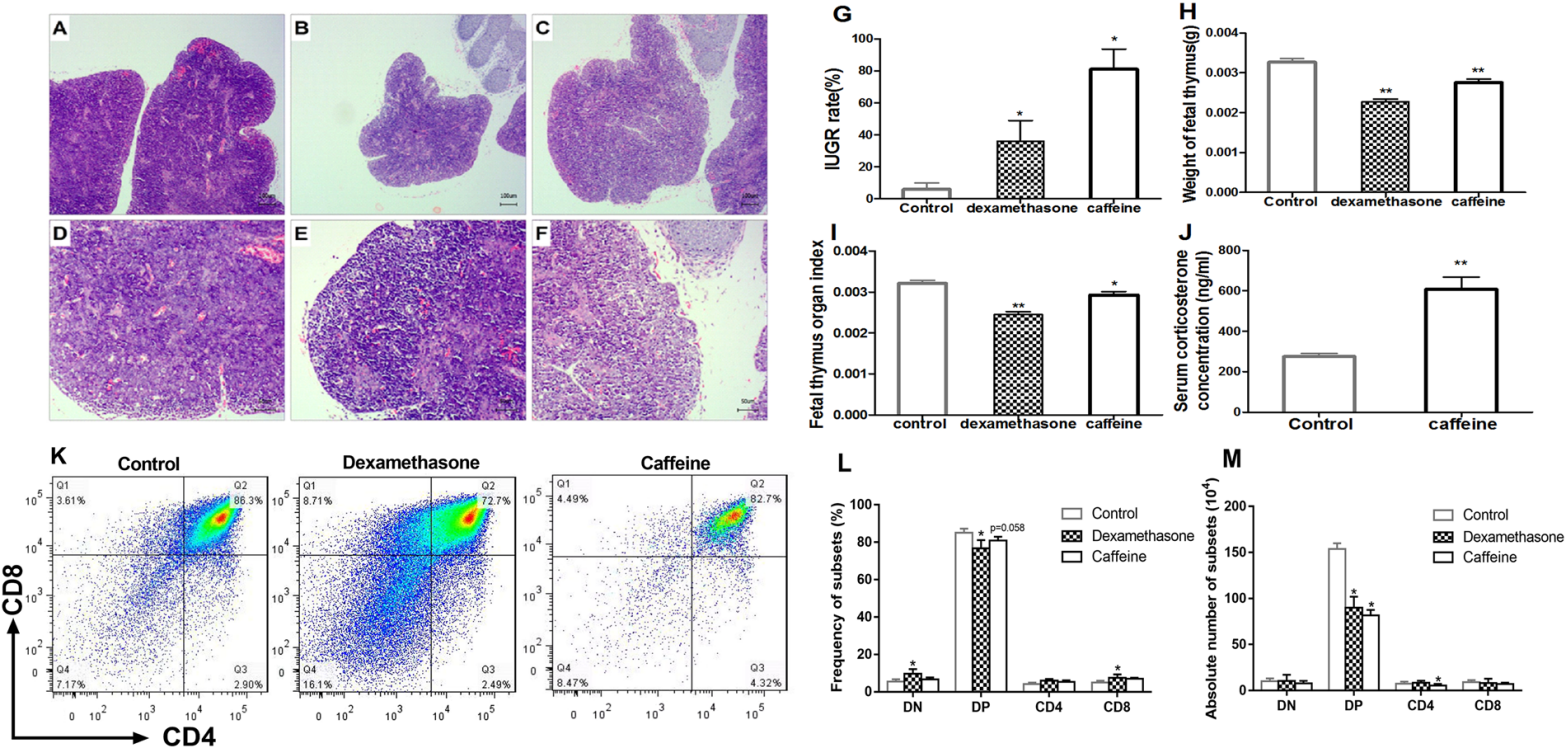
Effects of maternal caffeine and dexamethasone exposure on fetal thymus histological changes, fetal IUGR rates, fetal thymus development parameters and fetal thymocyte phenotypes on gestational day (GD) 18. (**A**) Control group (HE, ×40); (**B**) Dexamethasone group (HE, ×40); (**C**) Caffeine group (HE, ×40); (**D**) Control group (HE, ×100); (**E**) Dexamethasone group (HE, ×100); (**F**) Caffeine group (HE, ×100); (**G**) IUGR rates; (**H**) Thymus weights of the fetuses; (**I**) Fetal thymus organ index; (**J**) Fetal serum GC concentrations; (**K**) Typical flow diagram; (**L**) Percentage of thymocyte populations in the fetal mice; (**M**) Absolute thymocyte subpopulation numbers in the fetal thymuses. Mean ± SD, n = 3 per group for the flow cytometry analysis, n = 8–10 per group for the body weight and serum GC detection. *P < 0.05, **P < 0.01 *vs* control.

To confirm that caffeine treatment can induce GC elevation in fetal serum, we detected the GC concentration in fetal serum on gestational day (GD) 18. Consistent with our previous data (in rat models), maternal caffeine treatment significantly increased the serum GC level in the fetuses (*P* < 0.01, Fig. 5J) compared with the controls. To establish a typical intrauterine GC-overexposure model, dexamethasone was used as a positive control in our study. We observed significantly increased IUGR rates, decreased fetal thymus weights, and decreased organ index values in the caffeine and dexamethasone groups (*P* < 0.05*, P* < 0.01, Fig. 5G–I) compared to those of the controls.

### Histological changes in fetal thymus

Hematoxylin and eosin (HE) staining detection revealed that PCE could cause similar histological abnormalities in the fetal thymus as dexamethasone treatment. Compared with the controls (Fig. 5A and D), in both the dexamethasone (Fig. 5B and E) and caffeine (Fig. 5C and F) groups, there were more dilated intercellular spaces, greater narrowing of medulla, more red blood cell infiltration, and decreased numbers of thymocytes both in the cortex (immature thymocytes) and medulla (mature thymocytes).

### PCE altered thymocyte phenotypes in the fetus

To examine the effects of PCE on thymopoiesis in prenatal life, we analyzed the thymocyte phenotypes on GD 18. Compared with the controls, both the caffeine and dexamethasone treatments altered the thymocyte phenotypes in fetuses. As shown in Fig. 5L and M, the fetuses in the caffeine group exhibited higher percentages of DN, CD4SP and CD8SP cells, lower percentages of DP cells, and a lower absolute number of DP and CD4SP cells than the controls (*P* = 0.058, *P* < 0.05). Similar changes were observed in the dexamethasone group (*P* < 0.05, Fig. 5L and M).

### PCE increased thymocyte apoptosis in fetal mice

**Figure 6:**
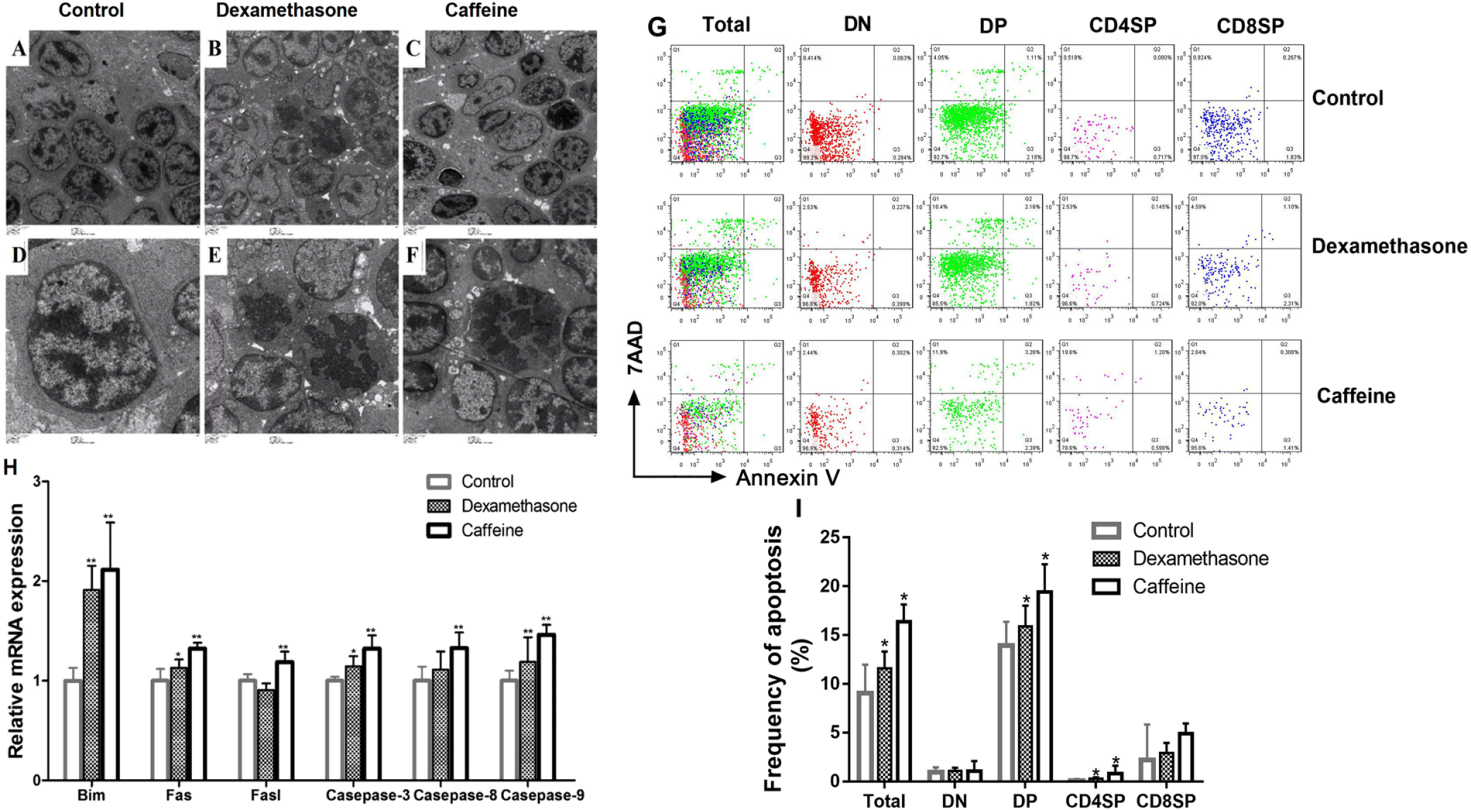
Fetal thymocyte apoptosis induced by maternal caffeine and dexamethasone exposure. (**A**) Control group (TEM, ×1,000); (**B**) Dexamethasone group (TEM, ×1,000); (**C**) Caffeine group (TEM, ×1,000); (**D**) Control group (TEM, ×3,000); (**E**) Dexamethasone group (TEM, ×3,000); (**F**) Caffeine group (TEM, ×3,000). (**G**) Typical flow diagram of thymocyte apoptosis; (**H**) mRNA expression levels of intrinsic and extrinsic apoptotic pathway genes; (**I**) Thymocytes apoptosis percentages in the fetal thymus. Mean ± SD, n = 3 per group for the flow cytometry analysis, n = 8–10 per group for the RT-PCR. *P < 0.05, **P < 0.01 *vs* control.

To examine whether the intrauterine GC elevation caused by PCE could induce fetal thymocyte apoptosis, transmission electron microscope (TEM) and flow cytometry analyses were used to examine thymocyte apoptosis. Under the TEM, more apoptotic cells and larger intercellular spaces in the fetal thymus were seen in the caffeine (Fig. 6C and F) and dexamethasone (Fig. 6B and E) groups compared with the controls (Fig. 6A and D). The apoptotic cells exhibited incomplete nuclear membranes, condensed chromatin, and nuclear fragmentation (Fig. 6E and F). Flow cytometry analysis also showed that the apoptotic percentages of the total DN and CD4SP thymocytes in the fetal thymuses of the caffeine mice were higher than those of controls (*P* < 0.05, Fig. 6G and I). Similar results were observed in the dexamethasone group (*P* < 0.05, Fig. 6G and I).

### PCE upregulated the mRNA expression of apoptotic signaling pathway genes in the fetal thymus

To determine whether the ‘intrinsic’ apoptotic pathway or the ‘extrinsic’ apoptotic pathway is involved in the PCE-mediated thymocyte apoptosis, we further detected the mRNA expression levels of both apoptotic pathways-related genes. Compared with the controls, the mRNA expression levels of Bim, Fas, and FasL, and all the downstream caspases (caspase-3, caspase-8, and caspase-9) in the fetal thymus of the caffeine group were significantly increased (*P* < 0.01, Fig. 6H). Caffeine exposure also significantly increased caspase-8 activity (*P* < 0.05, Fig. S1). Similarly, the mRNA expression of Bim, Fas, caspase-3, and caspase-9 in the fetal thymuses of the dexamethasone group was also significantly higher than the controls (*P* < 0.05, *P* < 0.01, Fig. 6H). Additionally, caspase-8 showed an increased tendency in the dexamethasone-exposed fetus.

## Discussion

In our previous studies, a clear dose-effect relationship between caffeine ingestion and changes of development-related parameters was observed after pregnant rats were treated with caffeine (20, 60, and 180 mg/kg/d). Additionally, the middle dose of caffeine at 60 mg/kg/d was the preferred dose to establish the IUGR rat model and to avoid excessive numbers of absorbed or stillborn fetuses^26,27^. According to the dose conversion (rat: mouse = 6.17: 12.33), the 60 mg/kg/d caffeine dose used in rats is equivalent to a 120 mg/kg/d caffeine dose for mice. By doing preliminary experiments, the caffeine dose (120 mg/kg/d) was reduced by eighty percent (96 mg/kg/d) to control the number of absorbed and stillborn fetuses in the present study. According to the World Health Organization, a caffeine intake of 300 mg/d in pregnant women is associated with increased IUGR risk^29^. Using the dose conversion between humans and mice (human: mouse 1:12.33)^30^, this dose of caffeine (96 mg/kg/d) is equivalent to a 467 mg/d consumption for a 60-kg pregnant woman, which is higher than 300 mg/d but less than the lowest dose usually needed to induce malformations in mice (100 mg/kg/d)^31,32^. Thus, the dose of caffeine used in present study should be reasonable. Mice embryos start implantation on GD 4.5 until GD 6.5, and the fetal thymus organogenesis is initiated on GD 10 and continues until birth^33,34^. Fetal thymocyte development is also well-established before GD 18^34^. Therefore, the caffeine exposure period from GD 9 to 18 in this study not only covered the periods of fetal thymus organogenesis and thymopoiesis, but also protected embryo implantation from the influence of caffeine. In this study, no miscarriage or stillborn fetuses were observed. In addition, the results showed that PCE significantly increased IUGR rates and decreased the fetal body weight, fetal thymus weight, and organ index values, which suggests that PCE can inhibit fetus and fetal thymus development.

Studies have shown that both estrogen and androgen can induce thymocyte apoptosis and influence immune responses, though the degrees of the pro-apoptotic effects from estrogen and androgen are different^35–37^. Therefore, to minimize the effects of hormones on the results of our study, we assessed the influence of PCE on the immune functions of female and male offspring separately. S. *pneumoniae* was chosen to establish the inflammation model in our study. S. *pneumoniae* is classified as a T cell-dependent antigen, which can activate Th cells and further mobilize B cells to produce IgG antibodies^38,39^. In S. *pneumoniae-*induced inflammation models, the IgG1/IgG2a ratio was used to reflect the tendency of distinct Th responses^40,41^. Briefly, the increase in the IgG1/IgG2a ratio is associated with an inhibited Th1 response and an upregulated Th2 response^42^. Some studies demonstrated that prenatal toxicants exposure could increase the IgG1/IgG2a ratio in offspring. Hong X *et al*. reported that maternal exposure to particulate matter could increase the IgG1/IgG2a ratio in offspring mice^43^. Additionally, Yamamoto S *et al*. reported that maternal exposure to 5 or 50 ppm toluene also resulted in increased serum IgG1 and decreased serum IgG2 concentrations in offspring mice^44^. Consistent with these studies, our data demonstrated that PCE reduced the serum IgG2 levels and obviously increased the IgG1 levels and the IgG1/IgG2a ratio in the offspring (before and after immunization). These findings suggest that PCE can alter the immune responses of offspring to pathogen challenge in postnatal life, as characterized by an attenuated Th1 response and an accelerated Th2 response.

The main representative effector cytokines of Th1 and Th2 cells are IFN-γ and IL-4, respectively. The expression of IFN-γ and IL-4 are used to reflect the Th1/Th2 balance^45^. Although both Th1 and Th2 cells are derived from thymocytes, their developmental patterns are different^46^. The skewing to Th2 during pregnancy is critical for fetal survival. Then, the increase in Th1 cell development after birth is needed to achieve the necessary Th1/Th2 balance^13^. Researchers found that failure to increase the Th1 number and diminish the capacity to mount IFN-γ-mediated Th1 responses due to prenatal adverse factors might be the main reason for the Th2 shift in adolescence^13,47–49^. Mainali *et al*. reported that perinatal dexamethasone exposure prevented increased Th1 levels and resulted in a Th1 inhibition phenotype in human offspring^50^. Maternal exposure to airborne particulate resulted in Th1 suppression and an increased IL-4/IFN-γ ratio in mouse offspring^43^. In our preliminary study, the serum IFN-γ concentration was below the minimum detection limit of the IFN-γ ELISA kit (5 pg/mL). Thus, we only detected the IL-4 content. In this study, we found that the serum IL-4 concentration and the splenic IL-4/IFN-γ ratio of the PCE offspring before S. *pneumoniae* immunization was significantly increased on PND 49. These findings suggest that PCE delays the postnatal Th1 increase and shapes a Th1 suppression status before antigen challenge, which might be the reason for the attenuated Th1 responses after immunization.

Fully functional thymopoiesis plays a key role in the maintenance of peripheral CD4^+^ naïve T cell numbers, which is critical for the Th1 increase in postnatal life^46^. Wang *et al*. found that maternal particulate matter exposure could lead to a reduction in peripheral CD4^+^ T cells and interrupted Th1 increase after birth in mouse offspring^51^. Veru F *et al*. reported that maternal stress could also reduce peripheral CD4^+^ lymphocytes in neonates and caused a Th1 inhibition immune phenotype in adolescents^14^. These findings suggest that decreased production of CD4^+^ naïve T cells during thymopoiesis might result in a failure to increase Th1 numbers after birth. Some studies indicated that prenatal exposure to immuno-toxicants, including heavy metals, diethylstilbestrol (DES), nicotine, dexamethasone, and tetrachlorodibenzo-p-dioxin could lead to impaired thymopoiesis in offspring through promoting thymocyte apoptosis^52–55^. Additionally, the different thymocyte subpopulations, at various stages of maturation and at different surface molecule expression levels, might be more or less susceptible to different immuno-toxicants. Prenatal nicotine exposure can not only increase thymocyte apoptosis through the Fas apoptotic pathway but can also promote CD4SP thymocyte apoptosis during negative selection^56^. DES showed stronger pro-apoptotic effects in fetal DN thymocytes (the most immature thymocyte) than in mature thymocytes^57^. GCs can exhibit pro-apoptotic effects on all thymocyte subsets. Among these subsets, DP cells are known to be the most sensitive subpopulation to GCs due to their low expression of the surface molecule, CD28^23^. All of them resulted in thymus atrophy and restricted thymopoiesis function in offspring. In the present study, PCE significantly increased apoptosis in total thymocytes and each thymocyte subpopulation on GD 18, PND 14 and PND 49. Additionally, the absolute number of DP and CD4SP cells was significantly decreased in the PCE group on GD 18, PND 14 and PND 49, which suggests that PCE can permanently impair CD4^+^ T cell thymopoiesis. Thymus atrophy and thymocyte apoptosis in the PCE group were also observed histologically. These results suggest that PCE can increase thymocyte apoptosis and alter thymocyte phenotypes, and finally attenuate thymopoiesis. Therefore, PCE might induce Th1 suppression by enhancing thymocyte apoptosis.

Our previous studies and other laboratories have reported that PCE could induce overexposure of the fetus to maternal GCs^26,58–60^. Definitely, GCs have pro-apoptotic effects on thymocyte in both humans and mice^19,21^. Prenatal synthetic GCs exposure or elevated GC levels in the fetus could also enhance thymocyte apoptosis^21,23^. Consistent with our previous results, PCE induced high GC levels in fetuses in this study. We speculated that the GC elevation might mediate the pro-apoptotic effects of PCE on thymocytes. To verify this hypothesis, we applied prenatal dexamethasone exposure as a positive control. The 0.5 mg/kg/d dexamethasone dose applied in this study is equal to 1/4 of the clinical dosage in human, which was used in our previous studies as a positive control^61^. In the present study, consistent with the dexamethasone group, the PCE mice showed thymus atrophy and similar patterns of thymocyte apoptosis. These findings support our hypothesis that PCE promotes thymocyte apoptosis by inducing maternal GC elevation in fetus.

During pregnancy, tightly regulated apoptosis of fetal thymocytes is crucial to establish functional thymopoiesis before birth^34^. In the different thymopoiesis stages, two distinct signaling pathways are involved in mediating thymocyte apoptosis. It was reported that the caspase-8-mediated extrinsic apoptotic pathway is utlized throughout the entire thymopoiesis process and mainly mediates the apoptosis of DN and DP cells^62^. However, Bim activation was the primary trigger for negative selection of developing thymocytes and mediates SP cell apoptosis^63^. It was reported that GCs could induce Fas expression in T cells and activate caspase-8-mediated extrinsic apoptosis during thymopoiesis^23^. Studies also reported that Bim was upregulated during GC induced apoptosis in T lymphoma cell lines^64,65^. Consistent with these findings, we found that the mRNA expression levels of Fas, Bim, and all the downstream caspases (caspase-3, caspase-8 and caspase-9) in the fetal thymus of both caffeine and dexamethasone treated groups were significantly increased compared to those of controls. These findings certified our hypothesis that PCE might induce maternal GC elevation in the fetus and thus promote DN, DP and SP cell apoptosis *via* the caspase-8-mediated extrinsic and Bim-regulated intrinsic apoptotic pathways. The synergistic effects of these two pathways promotes apoptosis in all thymocyte subpopulations and inhibits thymopoiesis.

## Conclusion

**Figure 7:**
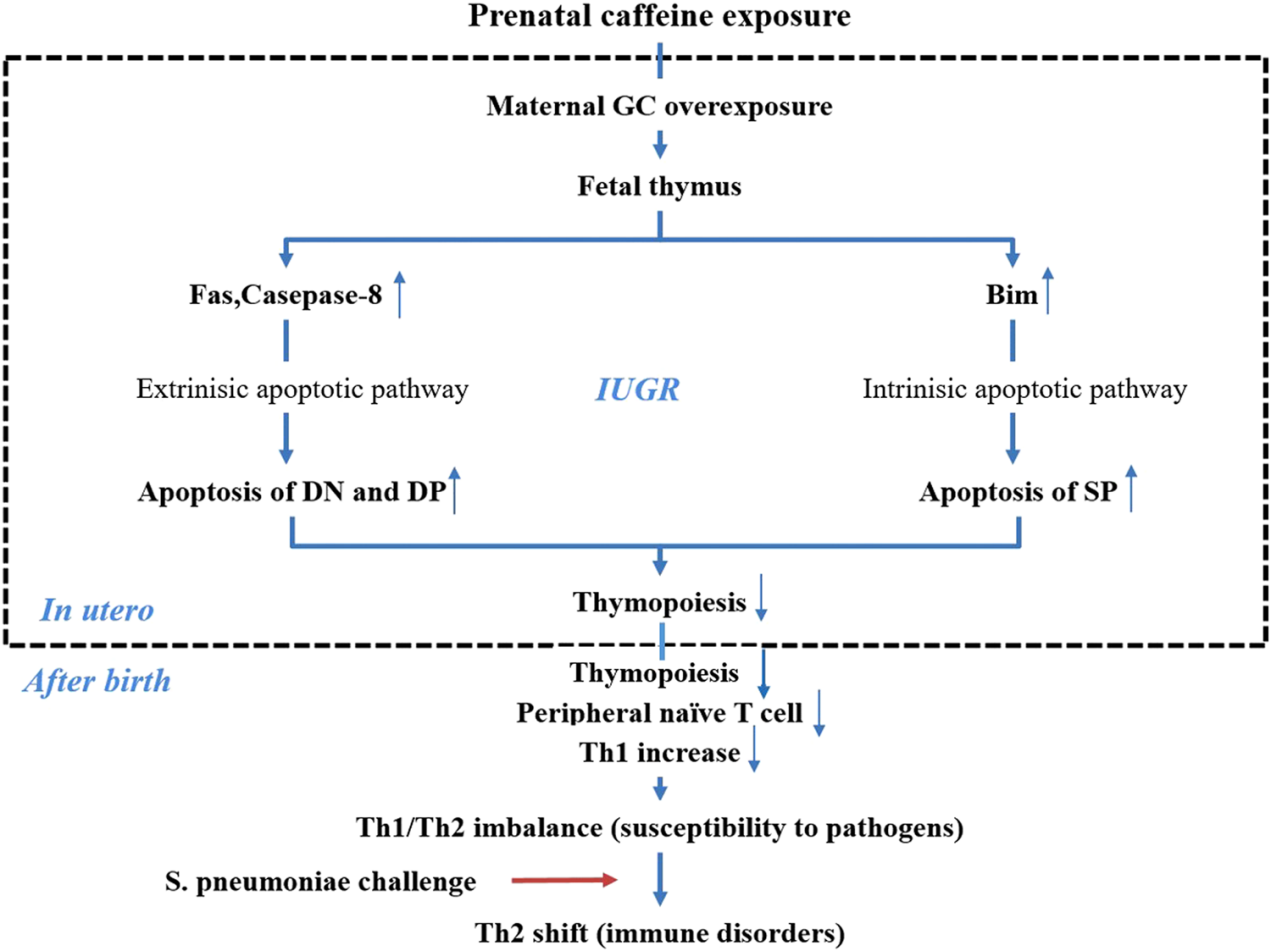
Hypothesis for the mechanism of the reduced thymopoiesis and Th1 inhibition in offspring induced by prenatal caffeine exposure (PCE). Bim, Bcl-2 family member; DN, CD4^−^CD8^−^ double negative thymocytes; DP, CD4^+^CD8^+^ double positive thymocytes; SP, single positive thymocytes; Th, T helper cells.

In summary, our study demonstrates that PCE has long-term toxic effects on fetal thymocyte development resulting in a deviation of the Th1/Th2 ratio before immunization, which attenuates Th1 responses after S. *pneumoniae* immunization. The underlying mechanism (Fig. 7) might be that PCE suppresses fetal thymopoiesis, which then impedes Th1 development in postnatal life by increasing thymocyte apoptosis. The elevation of maternal GCs caused by PCE is potentially responsible for the elevated apoptosis in the thymus by activating both the Fas-mediated and the Bim-regulated apoptotic pathways. Our study provides strong evidence for the connection between caffeine exposure during pregnancy and immune disorders in postnatal life, and is the first to demonstrate that PCE accelerates thymocyte apoptosis in the fetus and induces immune disorders in offspring.

## Materials and Methods

### Chemicals and reagents

Caffeine was purchased from Sigma-Aldrich Co., Ltd. (St. Louis, MO, USA). TRIzol was obtained from Invitrogen Co. (Carlsbad, CA, USA). The monoclonal antibodies (anti-mouse CD3-FITC, anti-mouse CD4-APC, anti-mouse CD8-PE-cy7, Rat IgG2b K Isotype Control FITC, Rat IgG2b K Isotype Control APC and Rat IgG2a K Isotype Control PE-cy7) and the Annexin V PE Apoptosis Detection kit were purchased from eBioscience (San Diego, USA). The mouse IgG1 and IgG2a enzyme-linked immunosorbent assay (ELISA) kits were purchased from MultiSciences (Hangzhou, Zhejiang, China). The mouse IL-4 ELISA kits were purchased from Dakewei Biotech (Shenzhen, Guangdong, China). The reverse transcription and real-time reverse-transcription PCR (RT-PCR) kits were obtained from Takara Biotechnology Co., Ltd. (Dalian, China). The other chemicals and reagents used were analytical grade.

### Animals and treatments

Mice of the Balb/C strain at 8-10 weeks were obtained from the Experimental Center of Hubei Medical Scientific Academy (No. 2008-0005, Wuhan, Hubei, China). Mice were maintained under specific pathogen-free conditions in the Center for Animal Experiment of Wuhan University (Wuhan, Hubei, China), which has been accredited by the Association for Assessment and Accreditation of Laboratory Animal Care International (AAALAC International). All procedures involving animals were performed according to Guidelines for the Care and Use of Laboratory Animals of the Chinese Animal Welfare Committee and were approved by the Committee on the Ethics of Animal Experiments at Wuhan University School of Medicine (Permit Number: 14016). The Balb/C strain of mice was used for this study due to its wide use in both immunology and developmental toxicity studies.

**Figure 8:**
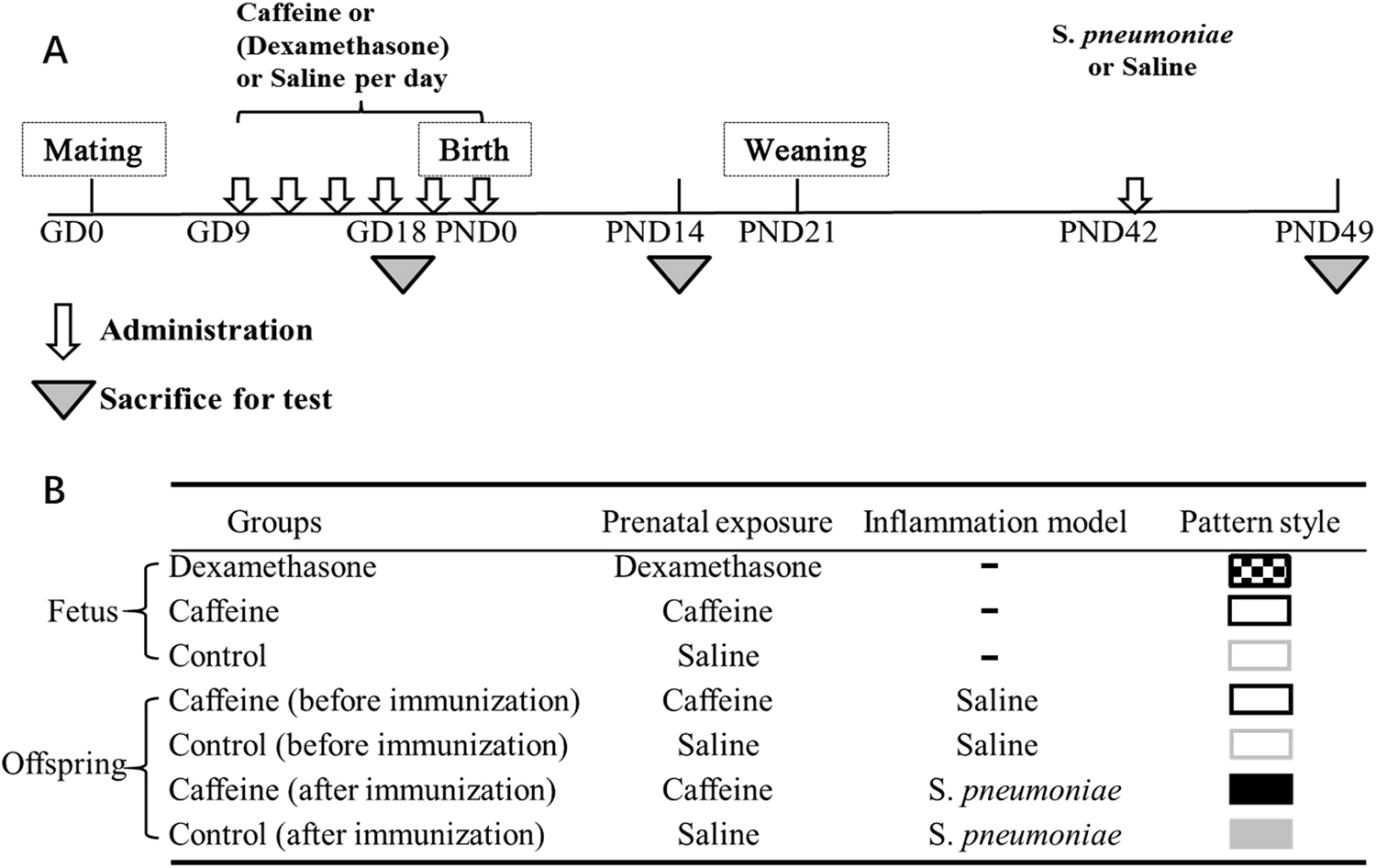
A schematic diagram of the protocol and groups. (**A**) Caffeine (dexamethasone) or saline was administered to pregnant mice from gestational day (GD) 9 to GD 18. On postnatal day (PND) 42, an inflammation model was established *via* S*. pneumoniae* immunization. Offspring were sacrifice for testing on GD 18, PND 14 and PND 49; (**B**) The experimental groups are shown in the table.

Mice were housed under standard conditions and allowed free access to standard food and water. A schematic of the maternal and offspring mice treatment procedures is shown in Fig. 8A and B. Briefly, after a one-week acclimation period, 2 females were placed with one male in a cage overnight for mating. Pregnancy was confirmed by the presence of a vaginal plug on the follow morning of cohabitation and this day was declared as GD 0. From GD 9 to GD 18, the pregnant mice were intraperitoneally administered 48 mg/kg of caffeine twice per day. Additionally, the pregnant mice in the dexamethasone group, which were used as the positive control of the PCE intrauterine mechanism, were intraperitoneally administered 0.25 mg/kg of dexamethasone twice per day to establish a typical GC-overexposure-induced IUGR model. The control group received the same volume of saline in the same frequency and intervals. On GD 18, the pregnant mice were sacrificed under isoflurane anesthesia and then fetuses from 8–10 litters (each litter 6–8 fetuses) per group were obtained and weighed. 3 Fetal thymuses per group were randomly selected for fixing in a phosphate-buffered 10% neutral formalin solution or 2.5% glutaraldehyde solution for HE staining and TEM analysis, respectively. The remaining fetal thymuses of the littermates were pooled together as one sample. Fetal serum, which was prepared from blood samples, from the same litter was also pooled into one sample. All samples were immediately frozen in liquid nitrogen and then stored at -80 °C for further analysis. In addition, 3 pooled thymus samples per group were used for flow cytometry detection as a three-time repeated experiment.

For the experiment with the offspring mice, the pregnant mice in the caffeine and control groups were kept until normal delivery (GD 19). On PND 0, the numbers of infants were normalized to 6 per dam to assure adequate and standardized nutrition. On PND 14, one pup per litter was selected randomly from 3 different mothers in each group per gender and euthanized for flow cytometry detection. On PND 21, the male and female offspring were separated and weaned. When the offspring matured, half of the offspring (8–10 mice per group per gender) were subcutaneously administered a *S. pneumoniae* vaccine to establish an inflammation model on PND 42. Then, the animals were anesthetized with isoflurane and sacrificed to collect blood samples 7 days later. Spleen samples were dissected and stored at -80 °C for subsequent analysis. Thymuses of 3 offspring per group per gender were harvested for flow cytometry. Body weight of the offspring was measured weekly, and the corresponding body weight gain rate was calculated as follows: Body weight gain rate (%) = [(body weight of PND × body weight of PND 7)]/body weight of PND 7 × 100%.

### Thymocyte staining and flow cytometry

Thymus was cut into small pieces and gently mashed through a 40 μm stainless steel sieve screen. Single cells were obtained by centrifugation at 335 × g for 5 min at 4 °C. Cells were re-suspended at 1 × 10^7^ cells/mL in FACS buffer (PBS, 1% FCS, 0.02% sodium azide) and then stained with 100 μL of antibody cocktail in FACS buffer (FITC-CD3, APC-CD4 and PE-cy7-CD8) for 30 min on ice in the dark. After surface staining, the cells were washed and incubated with 100 μL of fluorochrome-conjugated annexin V at room temperature for 10 min. Then, the cells were washed and re-suspended in 200 μL binding buffer. 7-aminoactinomycin D (7-AAD) was added before the flow cytometry analysis. The samples were processed with a BD FACS AriaTM III flow cytometer (BD Biosciences). Flow data were analyzed and plotted using the FlowJo software. The detection was repeated three times.

### Fetal thymus histopathology

For HE staining, the fixed fetal thymus tissues were dehydrated and then processed *via* the paraffin slice technique. Sections approximately 3 μm thick were stained with HE and observed under light microscope (100×, Olympus BX-53). For TEM analysis, the fixed fetal thymus samples were post-fixed with 1% osmium tetroxide and dehydrated through a graded series of ethanol, then embedded in EPON812. Ultrathin sections approximately 70 nm thick were cut on an EM UC7 ultramicrotome (Leica, Wetzlar, Germany) using a diamond knife. The sections were then dually stained with uranyl acetate and lead citrate, and then examined using a HT-7700 transmission electron microscope (Hitachi, Tokyo, Japan).

### RNA extraction and real-time RT-PCR

Total RNA was isolated from fetal thymus and offspring spleens using TRIzol reagent following the manufacturer’s protocol. Single-strand cDNA was prepared from 1 μg of total RNA using a reverse transcription kit. The primer sequences used and the annealing conditions for each gene are shown in Supplementary Table S1. PCR was performed in 96-well optical reaction plates using the QuantStudio 6 Flex from Applied Biosystems System (Foster City, CA, USA) in a total volume of 20 μL containing: 1 μL of cDNA template, 0.4 μL of each primer, 10 μL of 2× SYBRGreen, 0.4 μL of 50× ROX, and 7.8 μL of DEPC-H_2_O. The PCR cycling conditions used were as follows: pre-denaturation at 95 °C for 30 s and denaturation at 95 °C for 5 s. The annealing conditions for each gene are listed in Supplementary Table S1. Expression levels for each gene were calculated with the _ΔΔ_Ct method and normalized based on the glyceraldehyde-3-phosphate dehydrogenase (GAPDH) mRNA content. All primer sequences shown in Supplementary Table S1 were designed with Primer Premier 5.0 from PREMIER Biosoft International (Palo Alto, CA, USA) and queried with the NCBI BLAST database for homology comparison.

### Total IgG1 and IgG2a antibody and IL-4 measurements in the serum on PND 49

Serum IL-4, IgG1 and IgG2a contents were assayed by using ELISA kits following the manufacturer’s protocol. Undiluted serum was used for IL-4 detection. Additionally, the serum was diluted (100 μL) when the detecting serum IgG1 and IgG2a contents. The dilution ratio of the serum samples for the IgG1 detection was 1:10,000 and for IgG2a, it was 1:2,000. The optical density was read at 450 nm with a microplate reader.

### Statistical analyses

3 pooled fetal thymus samples per group and 3 offspring thymuses per group per gender were used for the flow cytometry analyses as three-time repeated experiments. For other experiments, the sample size (n) was 8-10 per group per gender for offspring and 8-10 per group for fetuses. All measurement data were presented as the mean ± SD. Differences between two groups were compared using *t*-tests, and differences among multiple groups were compared using analysis of variance (ANOVA). Values of *P* < 0.05 were considered statistically significant. Data were analyzed using SPSS 17 (SPSS Science Inc., Chicago, Illinois) and Prism (GraphPad Software, Inc., La Jolla, CA, USA, version 5.0).

## Electronic supplementary material

supplementary data

## References

[1] Dorostghoal M, Erfani Majd N, Nooraei P (2012). Maternal caffeine consumption has irreversible effects on reproductive parameters and fertility in male offspring rats. Clin Exp Reprod Med..

[2] Heckman MA, Weil J, Gonzalez de Mejia E (2010). Caffeine (1, 3, 7-trimethylxanthine) in foods: a comprehensive review on consumption, functionality, safety, and regulatory matters. J Food Sci..

[3] Kuczkowski KM (2009). Caffeine in pregnancy. Arch Gynecol Obstet..

[4] Grosso LM (2001). Maternal caffeine intake and intrauterine growth retardation. Epidemiology.

[5] Ping J (2014). Prenatal caffeine ingestion induces aberrant DNA methylation and histone acetylation of steroidogenic factor 1 and inhibits fetal adrenal steroidogenesis. Toxicology..

[6] Wu DM (2015). Increased DNA methylation of scavenger receptor class B type I contributes to inhibitory effects of prenatal caffeine ingestion on cholesterol uptake and steroidogenesis in fetal adrenals. Toxicol Appl Pharmacol..

[7] Sonnenschein-van der Voort AM (2016). Foetal and infant growth patterns, airway resistance and school-age asthma. Respirology..

[8] Xu XF (2014). Epigenetics of hyper-responsiveness to allergen challenge following intrauterine growth retardation rat. Respir Res..

[9] Verrill L, Bruns R, Luccioli S (2015). Prevalence of self reported food allergy in US adults: 2001, 2006, and 2010. Allergy Asthma Proc..

[10] Rees F (2016). The incidence and prevalence of systemic lupus erythematosus in the UK, 1999-2012. Ann Rheum Dis..

[11] Maio S (2016). Respiratory symptoms/diseases prevalence is still increasing: a 25-yr population study. Respir Med..

[12] Abrahamsson TR, Sandberg Abelius M, Forsberg A, Bjorksten B, Jenmalm MC (2011). A Th1/Th2-associated chemokine imbalance during infancy in children developing eczema, wheeze and sensitization. Clin Exp Allergy..

[13] McFadden JP, Thyssen JP, Basketter DA, Puangpet P, Kimber I (2015). T helper cell 2 immune skewing in pregnancy/early life: chemical exposure and the development of atopic disease and allergy. Br J Dermatol..

[14] Veru F, Dancause K, Laplante DP, King S, Luheshi G (2015). Prenatal maternal stress predicts reductions in CD4+ lymphocytes, increases in innate-derived cytokines, and a Th2 shift in adolescents: Project Ice Storm. Physiol Behav..

[15] Hoeman CM (2013). Developmental expression of IL-12Rbeta2 on murine naive neonatal T cells counters the upregulation of IL-13Ralpha1 on primary Th1 cells and balances immunity in the newborn. J Immunol..

[16] Miller JF (2002). The discovery of thymus function and of thymus-derived lymphocytes. Immunol Rev..

[17] Hughes GC, Clark EA, Wong AH (2013). The intracellular progesterone receptor regulates CD4+ T cells and T cell-dependent antibody responses. J Leukoc Biol..

[18] Dietert RR, Piepenbrink MS (2006). Perinatal immunotoxicity: why adult exposure assessment fails to predict risk. Environ Health Perspect..

[19] Bommhardt U, Beyer M, Hunig T, Reichardt HM (2004). Molecular and cellular mechanisms of T cell development. Cell Mol Life Sci..

[20] Kowalik A (2013). Dexamethasone-FITC staining application for measurement of circadian rhythmicity of glucocorticoid receptor expression in mouse living thymocyte subsets. J Neuroimmunol..

[21] Sacedon R (2000). Role of glucocorticoids in early T-cell differentiation. Ann N Y Acad Sci..

[22] Tuckermann JP, Kleiman A, McPherson KG, Reichardt HM (2005). Molecular mechanisms of glucocorticoids in the control of inflammation and lymphocyte apoptosis. Crit Rev Clin Lab Sci..

[23] Herold MJ, McPherson KG, Reichardt HM (2006). Glucocorticoids in T cell apoptosis and function. Cell Mol Life Sci..

[24] Yi J (2016). *In vivo* protective effect of betulinic acid on dexamethasone induced thymocyte apoptosis by reducing oxidative stress. Pharmacol Rep..

[25] Balcan E (2016). Quantitative approach to lectin-based glycoprofiling of thymic tissues in the control- and the dexamethasone-treated mice. Tissue Cell..

[26] Xu D (2012). Caffeine-induced activated glucocorticoid metabolism in the hippocampus causes hypothalamic-pituitary-adrenal axis inhibition in fetal rats. PLoS One..

[27] Liu Y (2012). Fetal rat metabonome alteration by prenatal caffeine ingestion probably due to the increased circulatory glucocorticoid level and altered peripheral glucose and lipid metabolic pathways. Toxicol Appl Pharmacol..

[28] Pettenuzzo LF (2008). Effects of chronic administration of caffeine and stress on feeding behavior of rats. Physiol Behav..

[29] Guilbert JJ (2003). The world health report 2002 - reducing risks, promoting healthy life. Educ Health..

[30] Brown N, Nagarkatti M, Nagarkatti PS (2006). Induction of apoptosis in murine fetal thymocytes following perinatal exposure to diethylstilbestrol. Int J Toxicol..

[31] Thayer PS, Palm PE (1975). A current assessment of the mutagenic and teratogenic effects of caffeine. CRC Crit Rev Toxicol..

[32] Koren G (2000). Caffeine during pregnancy? In moderation. Canadian family physician Medecin de famille canadien.

[33] Rossant J (2004). Lineage development and polar asymmetries in the peri-implantation mouse blastocyst. Semin Cell Dev Biol..

[34] Gordon J, Manley NR (2011). Mechanisms of thymus organogenesis and morphogenesis. Development..

[35] Barnard A (2008). Impact of the neuroendocrine system on thymus and bone marrow function. Neuroimmunomodulation..

[36] Zoller AL, Kersh GJ (2006). Estrogen induces thymic atrophy by eliminating early thymic progenitors and inhibiting proliferation of beta-selected thymocytes. J Immunol..

[37] Dulos GJ, Bagchus WM (2001). Androgens indirectly accelerate thymocyte apoptosis. Int Immunopharmacol..

[38] van de Beek D, de Gans J, Tunkel AR, Wijdicks EF (2006). Community-acquired bacterial meningitis in adults. N Engl J Med..

[39] Elhaik Goldman S (2016). Streptococcus pneumoniae fructose-1,6-bisphosphate aldolase, a protein vaccine candidate, elicits Th1/Th2/Th17-type cytokine responses in mice. Int J Mol Med..

[40] Xu J (2011). Intranasal vaccination with chitosan-DNA nanoparticles expressing pneumococcal surface antigen a protects mice against nasopharyngeal colonization by Streptococcus pneumoniae. Clin Vaccine Immunol..

[41] Vadesilho CF (2012). Characterization of the antibody response elicited by immunization with pneumococcal surface protein A (PspA) as recombinant protein or DNA vaccine and analysis of protection against an intranasal lethal challenge with Streptococcus pneumoniae. Microb Pathog..

[42] Abbas AK, Murphy KM, Sher A (1996). Functional diversity of helper T lymphocytes. Nature.

[43] Hong X (2013). Maternal exposure to airborne particulate matter causes postnatal immunological dysfunction in mice offspring. Toxicology.

[44] Yamamoto S (2009). Suppression of Th1- and Th2-type immune responses in infant mouse spleen after prenatal and postnatal exposure to low-level toluene and peptidoglycan. Inhal Toxicol..

[45] Zhu J, Paul WE (2008). CD4 T cells: fates, functions, and faults. Blood..

[46] Janson PC, Winerdal ME, Winqvist O (2009). At the crossroads of T helper lineage commitment-Epigenetics points the way. Biochim Biophys Acta..

[47] Seymour BW, Pinkerton KE, Friebertshauser KE, Coffman RL, Gershwin LJ (1997). Second-hand smoke is an adjuvant for T helper-2 responses in a murine model of allergy. J Immunol..

[48] Xiao R (2013). In utero exposure to second-hand smoke aggravates the response to ovalbumin in adult mice. Am J Respir Cell Mol Biol..

[49] Martino DJ, Prescott SL (2010). Silent mysteries: epigenetic paradigms could hold the key to conquering the epidemic of allergy and immune disease. Allergy..

[50] Mainali ES, Kikuchi T, Tew JG (2005). Dexamethasone inhibits maturation and alters function of monocyte-derived dendritic cells from cord blood. Pediatr Res..

[51] Wang P, You D, Saravia J, Shen H, Cormier SA (2013). Maternal exposure to combustion generated PM inhibits pulmonary Th1 maturation and concomitantly enhances postnatal asthma development in offspring. Part Fibre Toxicol..

[52] Hanson ML, Brundage KM, Schafer R, Tou JC, Barnett JB (2010). Prenatal cadmium exposure dysregulates sonic hedgehog and Wnt/beta-catenin signaling in the thymus resulting in altered thymocyte development. Toxicol Appl Pharmacol..

[53] Dietert RR, Lee JE, Hussain I, Piepenbrink M (2004). Developmental immunotoxicology of lead. Toxicol Appl Pharmacol..

[54] Lai ZW, Fiore NC, Hahn PJ, Gasiewicz TA, Silverstone AE (2000). Differential effects of diethylstilbestrol and 2,3,7,8-tetrachlorodibenzo-p-dioxin on thymocyte differentiation, proliferation, and apoptosis in bcl-2 transgenic mouse fetal thymus organ culture. Toxicol Appl Pharmacol..

[55] Cohen O, Ish-Shalom E, Kfir-Erenfeld S, Herr I, Yefenof E (2012). Nitric oxide and glucocorticoids synergize in inducing apoptosis of CD4(+)8(+) thymocytes: implications for ‘Death by Neglect’ and T-cell function. Int Immunol..

[56] Chen T (2016). Increased fetal thymocytes apoptosis contributes to prenatal nicotine exposure-induced Th1/Th2 imbalance in male offspring mice. Sci Rep..

[57] Besteman EG, Zimmerman KL, Holladay SD (2005). Diethylstilbestrol (DES)-induced fetal thymic atrophy in C57BL/6 mice: inhibited thymocyte differentiation and increased apoptotic cell death. Int J Toxicol..

[58] Kou H (2014). Maternal glucocorticoid elevation and associated blood metabonome changes might be involved in metabolic programming of intrauterine growth retardation in rats exposed to caffeine prenatally. Toxicol Appl Pharmacol..

[59] Luo H (2014). Prenatal caffeine ingestion induces transgenerational neuroendocrine metabolic programming alteration in second generation rats. Toxicol Appl Pharmacol..

[60] Xu D (2012). A hypothalamic-pituitary-adrenal axis-associated neuroendocrine metabolic programmed alteration in offspring rats of IUGR induced by prenatal caffeine ingestion. Toxicol Appl Pharmacol..

[61] Xu D, Chen M, Pan XL, Xia LP, Wang H (2011). Dexamethasone induces fetal developmental toxicity through affecting the placental glucocorticoid barrier and depressing fetal adrenal function. Environ Toxicol Pharmacol..

[62] Pozzesi N (2014). Role of caspase-8 in thymus function. Cell Death Differ..

[63] Bouillet P (2002). BH3-only Bcl-2 family member Bim is required for apoptosis of autoreactive thymocytes. Nature.

[64] Abrams MT, Robertson NM, Yoon K, Wickstrom E (2004). Inhibition of glucocorticoid-induced apoptosis by targeting the major splice variants of BIM mRNA with small interfering RNA and short hairpin RNA. J Biol Chem..

[65] Wang Z, Malone MH, He H, McColl KS, Distelhorst CW (2003). Microarray analysis uncovers the induction of the proapoptotic BH3-only protein Bim in multiple models of glucocorticoid-induced apoptosis. J Biol Chem..

