# Supplementary material for: Maternal Glucocorticoid Elevation and Associated Fetal Thymocyte Apoptosis are Involved in Immune Disorders of Prenatal Caffeine Exposed Offspring Mice
Source: Sci Rep. 2017 Oct 23;7:13746. doi: 10.1038/s41598-017-14103-7 (PMC5653827; doi:10.1038/s41598-017-14103-7)
Supplement: Supplementary file 1 — supplementary data [file 41598_2017_14103_MOESM1_ESM.doc]

**Title page**

**Maternal Glucocorticoid Elevation and Associated Fetal Thymocyte Apoptosis Are Involved in Immune Disorders of Prenatal Caffeine Exposed Offspring Mice**

**Han-xiao Liu, Ting Chen, Xiao Wen, Wen Qu, Sha Liu, Hui-yi Yan, Li-fang Hou & Jie Ping***

*Department of Pharmacology, Wuhan University School of Basic Medical Sciences, Wuhan 430071, China.*

***Corresponding author: Jie Ping**

Address: 185, East Lake Road, Wuhan 430071, China.

Tel.: +86 27 6875 8665.

Fax: +86 27 8733 1670.

E-mail: [pingjie@whu.edu.cn](mailto:pingjie@whu.edu.cn)

**Supplementary Table 1**

Supplementary Table 1. Oligonucleotide primers and PCR conditions of mouse in quantitative real-time PCR.

| *Genes* | *Forward primer* | *Reverse primer* | *Product （bp）* | *Annealing* |
| --- | --- | --- | --- | --- |
| Bim | CCGGAGATACGGATTGCACAG | CAGCCTCGCGGTAATCATTTG | 97 | 60°C,30 s |
| Caspase-3 | CGTGGTTCATCCAGTCCCTTT | ATTCCGTTGCCACCTTCCT | 102 | 60°C,30 s |
| Caspase-8  Caspase-9 | AGGTACTCGGCCACAGGTTA  TTCCCAGGTTTTGTCTCCTG | TGGGATGTAGTCCAAGCACA  CCTTTCGCAGAAACAGCATT | 137  143 | 60°C,30 s  60°C,30 s |
| Fas | ATGCACACTCTGCGATGAAG | CAGTGTTCACAGCCAGGAGA | 120 | 60°C,30 s |
| FasL | GCAGAAGGAACTGGCAGAAC | TTAAATGGGCCACACTCCTC | 128 | 60°C,30 s |
| GAPDH | AACTTTGGCATTGTGGAAGG | GGATGCAGGGATGATGTTCT | 132 | 60°C,30 s |
| IFN-γ | CTCAAGTGGCATAGATGTGGAAG | GATGGCCTGATTGTCTTTCAAG | 120 | 60°C,30 s |
| IL-4 | ATGGATGTGCCAAACGTCCT | AAGCACCTTGGAAGCCCTAC | 78 | 58°C,30 s; |

**Supplementary Figure 1**


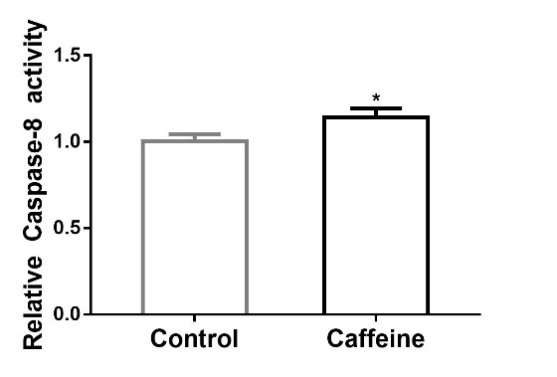


**Supplementary Figure 1. Effects of caffeine exposure on caspase-8 activity in fetal thymus.** Caspase-8 activity was determined using the Caspase Activity kit. Briefly, Fetal thymus tissues were lysed, and then 10 μl lysates were incubated with 80 μl reaction buffer and 10 μl substrate at 37 °C for 4 h, and then measured with an ELISA reader at an absorbance of 405 nm. Mean ± SD, n=5. ^*^*P <* 0.05 *vs* control.
